# Heart‐Cutting Two‐Dimensional Liquid Chromatography‐Isotope Ratio Mass Spectrometry for Compound‐Specific *δ* ^13^C Analysis of Water‐Soluble B Vitamins in Complex Supplement Matrices

**DOI:** 10.1002/jssc.70419

**Published:** 2026-04-21

**Authors:** Sarah P. Rockel, Jacqueline Martiny, Maik A. Jochmann, Torsten C. Schmidt

**Affiliations:** ^1^ Instrumental Analytical Chemistry University of Duisburg‐Essen Essen Germany; ^2^ Centre for Water and Environmental Research (ZWU) University of Duisburg‐Essen Essen Germany; ^3^ MDS Holding GmbH & Co. KG Dortmund Germany

**Keywords:** dietary supplements, food authenticity, isotope ratio mass spectrometry, reversed‐phase chromatography, stable carbon isotope analysis

## Abstract

Compound‐specific stable carbon isotope analysis by one‐dimensional liquid chromatography‐isotope ratio mass spectrometry (LC‐IRMS) is fundamentally constrained by the requirement for fully aqueous mobile phases, which limits chromatographic selectivity and prevents effective separation of analytes from complex sample matrices. Here, a heart‐cutting two‐dimensional LC‐IRMS method (2D‐LC‐IRMS) is presented for the first compound‐specific *δ* ^13^C analysis of the water‐soluble vitamins B5 (pantothenic acid) and B9 (folic acid) in commercial dietary supplements and fortified beverages. An organic‐modified reversed‐phase separation in the first dimension achieved matrix reduction, while a fully aqueous second‐dimension separation ensured IRMS compatibility and delivered baseline‐resolved analyte peaks for precise isotope determination. Validation demonstrated linear calibration over 2–100 mgC L^−1^ with *R* ^2^ ≥ 0.9999, repeatability of ≤ 0.13‰, and isotope‐stability‐based method detection limits of 5 mgC L^−1^. Analyzed *δ* ^13^C values were independent of chromatographic configuration, confirming that heart‐cutting transfer does not introduce isotope fractionation. Application to 12 commercial products, including tablets, effervescent formulations, powdered supplements, and an energy drink, yielded *δ* ^13^C ranges of −20.6‰ to −32.9‰ for vitamin B5 and −20.4‰ to −36.4‰ for vitamin B9, reflecting differences in synthetic production routes across manufacturers. The presented workflow extends LC‐IRMS to the compound class of water soluble vitamins that were so far inaccessible to this technique and provides a broadly applicable strategy for compound‐specific isotope analysis of polar analytes in challenging matrices.

## 1 Introduction

Compound‐specific stable carbon isotope analysis (CSIA) is a powerful analytical tool for authenticity assessment, source attribution, and investigation of production pathways [1, 2, 3]. Liquid chromatography‐isotope ratio mass spectrometry (LC‐IRMS) enables isotope analysis of nonvolatile, polar, and thermally labile compounds that are not amenable to gas chromatography [4]. Despite its analytical potential, LC‐IRMS is subject to strict methodological constraints, most notably the requirement for fully aqueous mobile phases to avoid introduction of extraneous carbon into the isotope ratio measurement [5, 6]. This requirement substantially limits chromatographic selectivity and often prevents adequate separation of target analytes from complex sample matrices. The challenge is particularly pronounced for dietary supplements and fortified beverages, which contain highly variable compositions including sweeteners, organic acids, binders, caffeine, and multiple micronutrients [7, 8]. Under fully aqueous reversed‐phase conditions, coelution of matrix components is common, and certain hydrophobic formulation constituents may not elute at all, leading to persistent column contamination and deteriorating chromatographic performance.

Conventional liquid chromatographic methods for vitamin determination in dietary supplements typically employ organic solvents or buffered mobile phases and rely on UV, fluorescence, or mass spectrometric detection [9, 10]. While these approaches provide sufficient selectivity for concentration analysis, they are incompatible with IRMS detection and therefore unsuitable for CSIA measurements. In an early proof of principle study Caimi and Brenna employed a moving wire LC‐IRMS to analyze fat soluble vitamins [11]. However, to date, LC‐IRMS has not been applied to the isotope analysis of water soluble vitamins in commercial supplement matrices, largely due to these chromatographic and solvent‐related limitations.

Two‐dimensional liquid chromatography coupled to IRMS (2D‐LC‐IRMS) offers an effective strategy to overcome these constraints [12, 13]. In a heart‐cutting configuration, an organic‐modified reversed‐phase separation in the first dimension can be used to achieve efficient matrix reduction, while selective transfer of the analyte of interest into a second dimension operated under fully aqueous conditions ensures compatibility with isotope ratio detection. The second dimension thereby functions as a purification interface, isolating the analyte from organic solvent background and residual matrix components prior to combustion and isotope analysis.

Water‐soluble B vitamins represent analytically challenging model compounds for this approach. Pantothenic acid (vitamin B5) and folic acid (vitamin B9) are widely incorporated into multivitamin supplements and fortified beverages, resulting in highly heterogeneous matrices and broad concentration ranges [14]. Their polarity, combined with the complexity of formulation excipients, makes them suitable test cases for further evaluating the capabilities and limitations of 2D‐LC‐IRMS.

The aim of this study was to develop and validate a heart‐cutting 2D‐LC‐IRMS method for compound‐specific *δ* ^13^C analysis of vitamins B5 and B9 in complex dietary supplement and beverage matrices, and to assess its performance with respect to chromatographic selectivity, isotopic integrity, and applicability to real commercial products.

## 2 Materials and Methods

### 2.1 Chemicals and Standards

D‐pantothenic acid hemicalcium salt (vitamin B5, ≥ 99%) was purchased from Sigma‐Aldrich (St. Louis, USA). Folic acid dihydrate (vitamin B9, 97%) was obtained from Thermo Fisher Scientific (Waltham, USA). Certified isotope reference materials USGS40 (L‐glutamic acid, *δ* ^13^C = −26.39‰ VPDB) and USGS41a (L‐glutamic acid enriched in ^13^C, *δ* ^13^C = +36.55‰ VPDB) were purchased from IVA Analysentechnik GmbH (Meerbusch, Germany) and used for *δ* ^13^C normalization.

Orthophosphoric acid (H_3_PO_4_, ≥ 99%), sodium peroxodisulfate (Na_2_S_2_O_8_, ≥ 99%), and sulfuric acid (H_2_SO_4_, ≥ 98%) were obtained from Fluka (Steinheim, Germany). Methanol and acetonitrile (LC‐MS grade) were purchased from Merck KGaA (Darmstadt, Germany). Ultrapure water (resistivity ≥ 18.2 MΩ cm) was produced using an Arium Pro VF water purification system (Sartorius Stedim Biotech GmbH, Göttingen, Germany) and used for all aqueous solutions.

Oxidation reagents for the LC‐IRMS interface were prepared as follows: orthophosphoric acid was diluted to 1.5 mol L^−1^, and sodium peroxodisulfate was dissolved at a concentration of 100 mg L^−1^ in ultrapure water. Both solutions were degassed for 15 min under vacuum in an ultrasonic bath (Bandelin electronic GmbH & Co., Berlin, Germany, Vacuubrand GmbH & Co., Wertheim, Germany) and continuously purged with high‐purity helium (99.999%, Air Liquide, Paris, France) to minimize CO_2_ contamination prior to use. Stock standard solutions of vitamins B5 and B9 were prepared in ultrapure water at a concentration of 100 mgC L^−1^ (expressed as milligrams of carbon per liter), acidified with sulfuric acid to a pH of 3 and placed in an ultrasonic bath for 15 min (Bandelin electronic GmbH & Co., Berlin, Germany).

### 2.2 Sample Preparation

Twelve commercial dietary supplements and fortified beverages purchased in German drugstores and supermarkets were selected to represent a wide range of matrices commonly encountered in vitamin analysis. The samples included multivitamin tablets, vitamin B‐complex supplements, effervescent tablets, powdered drink supplements, and an energy drink. A detailed list of the analyzed products is provided in Table S1.

Sample preparation was adapted from manufacturer guidelines for the determination of water‐ and fat‐soluble vitamins by HPLC [15]. For solid supplements, tablets were finely ground using a mortar and pestle. An appropriate amount of each sample was weighed into volumetric flasks and extracted with ultrapure water. Extraction volumes ranged from 10 to 150 mL depending on sample type and the declared vitamin content, yielding final carbon concentrations suitable for LC‐IRMS analysis (30–100 mgC L^−1^). To prevent complexation of the target analytes by tablet excipients, the extracts were acidified with sulfuric acid to a final pH of approximately 3. The samples were subsequently placed in an ultrasonic bath (Bandelin electronic GmbH & Co., Berlin, Germany) for 15 min to enhance extraction efficiency. After sonication, the solutions were filtered through 0.45 µm syringe filters (cellulose acetate, 25 mm diameter; BGB Analytik AG, Böckten, Switzerland) prior to LC‐IRMS analysis. Liquid samples (energy drink) were analogously acidified and filtered prior to analysis. All sample extracts were analyzed immediately after preparation.

### 2.3 Instrumentation and Conditions

A 2D‐LC‐IRMS configuration was employed, building on the approach described by Rockel et al. [12]. The first chromatographic dimension was operated in reversed‐phase mode to achieve matrix separation using organic mobile phases, while the second dimension was operated under fully aqueous conditions compatible with IRMS detection.

#### 2.3.1 First‐Dimension LC Conditions

The first dimension was performed on a Vanquish UHPLC system (Thermo Fisher Scientific, Waltham, USA) equipped with a UV detector and column oven. Separation was achieved using an Acquity UPLC BEH C18 column (50 mm × 2.1 mm, 1.7 µm particle size; Waters, Milford, USA). The column temperature was maintained at 30°C using a Thermasphere TS‐130 column oven (Phenomenex, Torrance, USA). The injection volume was 10 µL. All samples were analyzed in triplicate. The mobile phase consisted of (A) ultrapure water acidified with sulfuric acid to pH 3 and (B) acetonitrile. The flow rate was 0.3 mL min^−1^, the applied gradient was as follows: 0–9 min, 0% B; 9–10 min, linear increase to 10% B; 10–21 min, 10% B; 21–23 min, linear increase to 40% B; 23–28 min, 40% B; 28–29 min, return to 0% B, followed by re‐equilibration until the end of the run at 40 min. UV detection in the first dimension was used to monitor analyte elution and to define heart‐cutting transfer windows. For vitamin B5, UV detection was performed at 190 and 210 nm; for vitamin B9, UV detection wavelengths were set to 254 and 280 nm.

#### 2.3.2 Heart‐Cutting and Transfer Conditions

Heart‐cutting transfer between the first and second chromatographic dimensions was performed using a two‐position six‐port switching valve (Rheodyne LLC, Bensheim, Germany) equipped with a 500 µL stainless steel sample loop. Switching windows were defined based on UV detection in the first dimension. In CSIA it is important, that the whole peak is transferred, to get accurate stable carbon isotope measurements [12]. For vitamin B5, the heart‐cutting transfer was performed between 7.5 and 9.0 min, corresponding to a transferred volume of approximately 450 µL. For vitamin B9, the transfer window was set between 13.6 and 14.4 min, resulting in a transferred volume of approximately 240 µL. Following transfer, the analytes were flushed from the loop onto the second‐dimension column using the fully aqueous mobile phase. For B9, an at‐column‐dilution [16] was employed using ultrapure water with a dilution factor of 2.5 to avoid solvent incompatibilities.

#### 2.3.3 Second‐Dimension LC and IRMS Conditions

The second dimension was operated on an Ultimate 3000 LC system (Thermo Fisher Scientific, Waltham, MA, USA) and coupled to a high‐temperature LC‐IRMS interface (IsoLink) connected to a Delta V isotope ratio mass spectrometer (Thermo Fisher Scientific, Bremen, Germany). Separation in the second dimension was performed using an YMC Triart C18 column (50 mm × 2.1 mm, 3 µm particle size; YMC Europe GmbH, Dinslaken, Germany). The mobile phase consisted of ultrapure water delivered at a flow rate of 0.5 mL min^−1^. The column oven of the Vanquish system of the first dimension was used for temperature control of the second‐dimension columns. For vitamin B5 the column temperature was maintained at 20°C. For vitamin B9, the column temperature was initially set to 30°C and increased to 60°C after 30 min after initial injection. The total runtime of the 2D‐method was 40 min.

Post‐column oxidation was performed using sodium peroxodisulfate and phosphoric acid solutions delivered at 50 µL min^−1^ each. The oxidation reactor was maintained at 100°C. CO_2_ reference gas peaks were introduced via open split two times in the beginning and three times in the end of each run. Standards and samples were subjected to identical chromatographic and IRMS conditions.

### 2.4 Alternative Chromatographic Configurations

Analyte breakthrough represents a potential concern in heart‐cutting 2D‐LC, when the transferred fraction contains organic modifier that is stronger than the initial mobile phase of the second dimension. Breakthrough experiments conducted with increasing ACN contents demonstrated that peak fronting became apparent at 10% ACN, with complete breakthrough at 30% ACN. For vitamin B5, the transfer is performed under 100% water in the first dimension, so breakthrough does not occur. For vitamin B9, where the transfer window falls within a region of 10% ACN content, at‐column‐dilution approach was employed to reduce the organic load of the transferred fraction, effectively preventing breakthrough effects. This strategy should be considered when applying the method to analytes eluting under higher organic modifier conditions.

A one‐dimensional LC‐IRMS setup operated under fully aqueous conditions was employed for comparison with the two‐dimensional approach. In these experiments separation was performed using the YMC Triart C18 column (50 mm × 2.1 mm, 3 µm particle size; YMC Europe GmbH, Dinslaken, Germany) at a temperature of 30°C. Injection volumes were 10 µL. The eluent was ultrapure water at 0.5 mL min, the interface conditions were identical to the ones described above.

Furthermore, experiments employing methanol (MeOH) as organic modifier in the first chromatographic dimension were performed with a mobile phase composition of Solvent A (ultrapure water acidified to pH 3 with sulfuric acid) and Solvent B (MeOH). The gradient program was as follows: 0–10.5 min, 0% B; 10.5–15.5 min, linear increase to 15% B; 15.5–23.5 min, 15% B; 23.5–26.5 min, linear increase to 100% B; 26.5–33.5 min, 100% B; 33.5–34.5 min, return to 0% B, followed by re‐equilibration until 40 min. The temperature of the second‐dimension column was set at 5°C in the beginning of the run and increased to 30°C after 26 min. All other instrumental parameters, including flow rate, column temperature of the first dimension, and IRMS operating conditions, were identical to those used for the optimized configuration unless stated otherwise.

Additional calibration experiments were conducted using an alternative reversed‐phase column in the second dimension (XBridge Premier BEH C18 (50 mm × 2.1 mm, 3.5 µm particle size, Waters Corporation, Milford, USA) to assess column‐dependent effects on isotope ratio measurements. All other instrumental parameters, including flow rates, oxidation conditions, and IRMS settings, were kept identical to those described above.

### 2.5 Isotope Referencing and Data Handling

Carbon isotope ratios are reported in *δ* ^13^C notation relative to the Vienna Pee Dee Belemnite (VPDB) scale. External calibration and normalization were performed using certified isotope reference materials previously characterized by elemental analyzer‐isotope ratio mass spectrometry (EA‐IRMS). EA‐IRMS analyses were conducted using a PYRO Cube elemental analyzer operated in C/N mode coupled to an IsoPrime 100 isotope ratio mass spectrometer (Elementar Analysensysteme GmbH, Langenselbold, Germany). Normalization of LC‐IRMS *δ* ^13^C values was performed using a two‐point calibration approach with the certified reference materials USGS40 and USGS41a, following established procedures described in the literature [1]. This approach ensures traceability of the *δ* ^13^C values to the international VPDB scale.

As previously reported for LC‐IRMS analyses, a systematic offset between normalized EA‐IRMS values and LC‐IRMS measurements may occur due to differences in oxidation and CO_2_ transfer processes [17, 18]. Such an offset was also observed in this study and was consistent across all measurements. As both standards and samples were treated identically, this offset does not affect the comparability or interpretation of the compound‐specific *δ* ^13^C values.

Acquisition and processing of LC‐IRMS data were performed using Isodat 3.0 software (Thermo Fisher Scientific, Bremen, Germany). Reference gas pulses were introduced at the beginning and at the end of each chromatographic run to ensure instrumental stability and accurate isotope ratio determination. All reported *δ* ^13^C values represent the mean of triplicate LC‐IRMS measurements.

### 2.6 Method Validation

#### 2.6.1 Calibration and Linearity

External calibration was performed using independently prepared standard solutions of vitamins B5 and B9 covering the concentration range of 2–100 mgC L^−1^. Calibration solutions were analyzed under the same chromatographic and instrumental conditions as samples. Linearity was assessed by plotting analyzed *δ* ^13^C values as a function of injected carbon amount and calculating the coefficient of determination (*R* ^2^) using least‐squares regression.

#### 2.6.2 Precision and Repeatability

Repeatability of isotope measurements was evaluated by triplicate injections of standards and selected samples within a single analytical sequence. Precision was expressed as the standard deviation (SD) of *δ* ^13^C values. Instrumental stability during runs was monitored by repeated injections of reference gas pulses at the beginning and end of each sequence. Stability was considered acceptable when reference gas *δ* ^13^C values and peak areas remained constant within analytical uncertainty (± 0.5‰).

#### 2.6.3 Method Detection Limits

Method detection limits (MDLs) were determined based on isotope ratio stability rather than signal‐to‐noise criteria, in accordance with common practice in CSIA [19]. The MDL was defined as the lowest concentration at which *δ* ^13^C values remained stable within ± 0.5‰ of the expected value. To determine MDLs, decreasing concentrations of standard solutions were analyzed and *δ* ^13^C values were evaluated using a moving mean approach. For each concentration level, a moving mean of consecutive measurements (*n* = 3) was calculated. The concentration at which the moving mean deviated by more than 0.5‰ from the reference value, or where the associated SD exceeded 0.5‰, was considered below the reliable quantification range. The MDL was defined as the lowest concentration above this threshold. This approach accounts for increased isotopic variability at low signal intensities and reflects the practical limit at which isotope ratios can be determined with acceptable precision.

#### 2.6.4 Robustness Assessment

Method robustness was evaluated by comparing *δ* ^13^C values obtained under different chromatographic configurations, including variation of first‐dimension organic modifier (acetonitrile vs. methanol), stationary phase, and dimensionality (1D vs. 2D). Agreement within analytical uncertainty (± 0.5‰) was interpreted as evidence of configuration‐independent isotope determination.

## 3 Results and Discussion

### 3.1 Comparison of One‐ and Two‐Dimensional LC‐IRMS for Matrix‐Rich Samples

**FIGURE 1:**
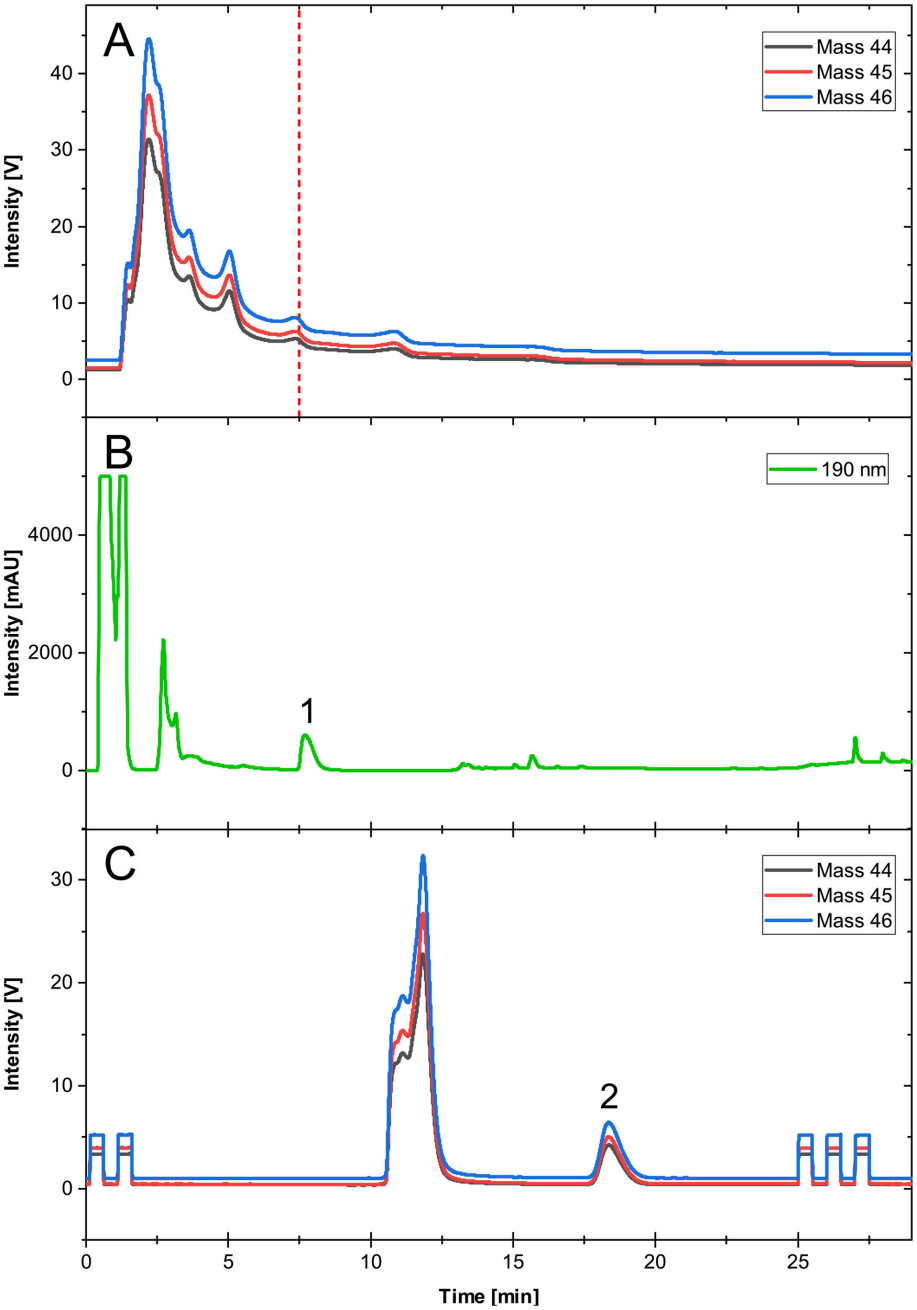
Comparison of one‐dimensional and two‐dimensional LC analysis of the same supplement sample containing vitamin B5. (A) Fully aqueous 1D LC‐IRMS chromatogram showing severe coelution. Red‐dashed line shows retention time of vitamin B5 under one‐dimensional LC‐IRMS conditions. (B) First‐dimension chromatogram at 190 nm for the detection of vitamin B5 (Peak no. 1) with ACN as organic modifier. (C) Fully aqueous second‐dimension chromatogram after heart‐cutting transfer demonstrating baseline‐resolved separation of vitamin B5 (Peak no. 2).

To evaluate whether conventional LC‐IRMS conditions are sufficient for CSIA of B vitamins in complex matrices, a representative dietary supplement sample (Sample 10) was analyzed using a fully aqueous one‐dimensional LC method compatible with IRMS detection. The same sample was used for all chromatographic experiments to ensure direct comparability. Under these conditions, numerous matrix components coeluted with each other resulting in severe signal overlap and distorted chromatographic profiles (Figure 1A). Because the analyte signals were superimposed with multiple unresolved matrix contributions and no stable baseline could be established, reliable peak integration was not possible and no accurate *δ* ^13^C value could be determined, as IRMS integrates the CO_2_ coming from all carbon atoms in an unresolved peak [20]. The retention time of vitamin B5 under one‐dimensional LC‐IRMS conditions was approximately 7.5 min (red dashed line, Figure 1A), as determined from prior standard injections.

Application of the heart‐cutting two‐dimensional LC approach enabled selective transfer of the analyte fraction (vitamin B5) from the first dimension to the second dimension. Both methanol and acetonitrile were evaluated as organic modifiers in the first chromatographic dimension. Acetonitrile resulted in reduced organic background transfer and improved peak shape in the second dimension and was used as the preferred method, although both approaches are in principle viable. A detailed comparison is provided in Figure S1. The first‐dimension chromatogram (Figure 1B) shows that vitamin B5 elutes at a retention time of 7.7 min. After heart‐cutting transfer, the second‐dimension chromatogram (Figure 1C) demonstrates baseline‐resolved separation of the analyte from residual background, yielding a symmetrical peak suitable for isotope ratio determination. Under these conditions, vitamin B5 eluted at 18.3 min in the second dimension.

Baseline carbon background levels before and after heart‐cutting transfer were comparable, indicating that improvements in chromatographic performance arise primarily from removal of coeluting matrix components rather than changes in instrumental background. Isotopic stability throughout each run was verified by repeated injections of reference gas pulses. The reference peaks showed excellent repeatability, with average SDs within a run of 0.05‰ for *δ* ^13^C values and 0.08 Vs for peak areas, confirming stable instrument performance.

These results demonstrate that matrix‐induced coelution represents a critical limitation for direct LC‐IRMS analysis of dietary supplements and highlight the necessity of two‐dimensional chromatographic purification prior to isotope ratio measurement. The heart‐cutting 2D‐LC configuration therefore functions not only as a separation strategy but also as an essential compatibility interface between complex sample matrices and IRMS detection requirements.

### 3.2 Resolution of Coeluting Matrix Components

**FIGURE 2:**
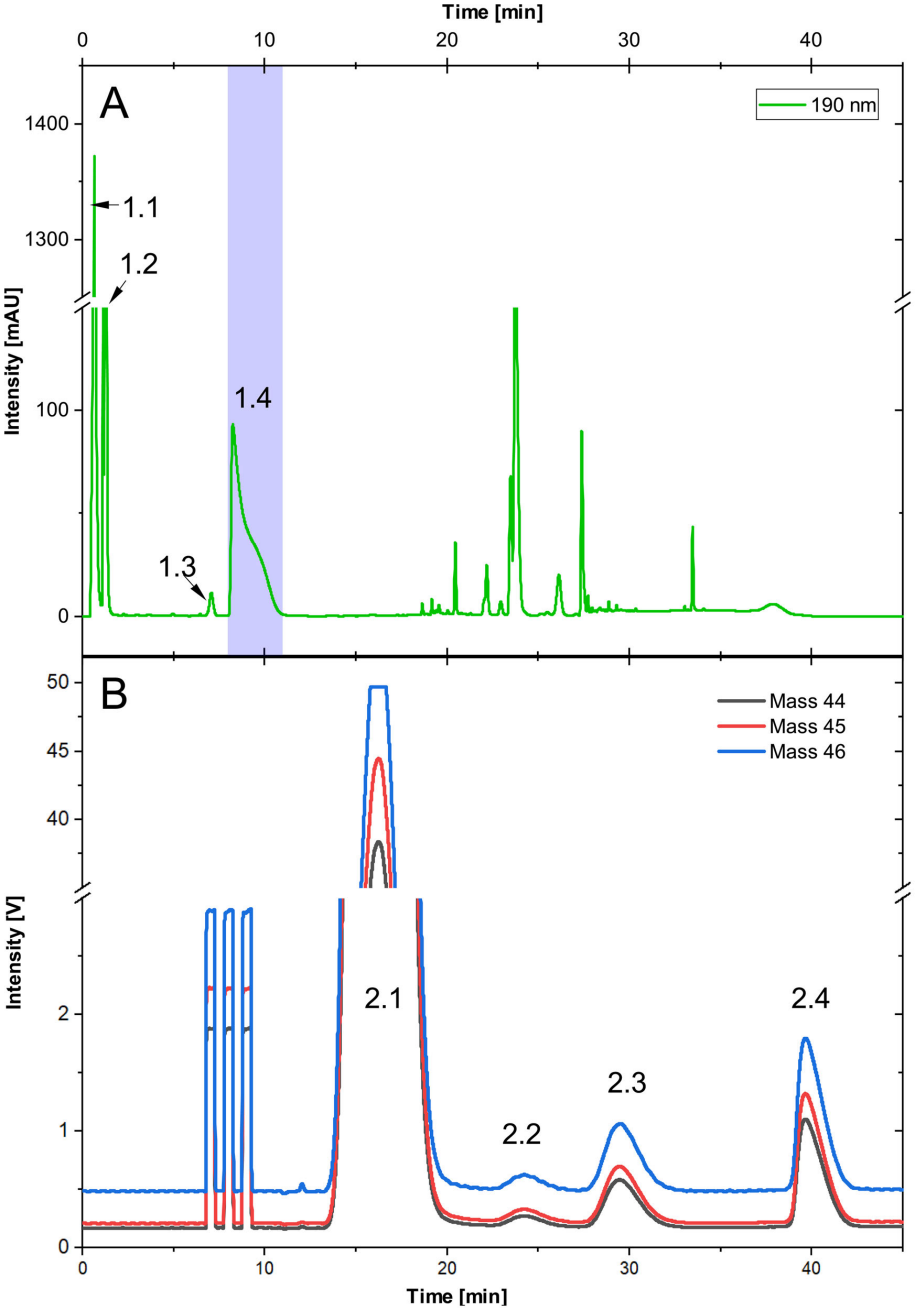
Resolution of coeluting compounds by heart‐cutting 2D‐LC. (A) First‐dimension chromatogram (UV, 190 nm) showing coelution of vitamin B5 with a matrix component in a multivitamin effervescent tablet extract. (B) Second‐dimension chromatogram (IRMS *m*/*z* 44, *m*/*z*45, and *m*/*z* 46) after transfer of the 8–11 min fraction demonstrating baseline separation.

Beyond enabling analyte isolation from complex sample matrices, the two‐dimensional LC approach provides the possibility to separate compounds that coelute in the first chromatographic dimension. This capability is illustrated using a representative multivitamin effervescent tablet sample containing vitamin B5 analyzed under first‐dimension methanol gradient conditions. The first‐dimension chromatogram recorded by UV detection at 190 nm (Figure 2A) shows that under these conditions the analyte elutes within a region where at least one additional component coelutes, as indicated by a pronounced peak shoulder (peak 1.4). Such composite peak profiles demonstrate incomplete chromatographic resolution and would prevent reliable analyte isolation if only a single separation dimension were used.

To ensure complete transfer of the analyte fraction, a comparatively wide heart‐cutting window from 8 to 11 min was selected (Figure 2A, blue box). At a first‐dimension flow rate of 0.3 mL min^−1^, this corresponds to a transferred volume of approximately 0.9 mL. For this purpose, a 1.2 mL sampling loop was employed to avoid sample loss during transfer. For separation of the coeluting compounds in the second dimension, a temperature gradient was employed, starting at 5°C and raising the temperature after 33 min to 30°C. Following heart‐cutting, second‐dimension analysis by IRMS (monitoring *m*/*z* 44, *m*/*z* 45, and *m*/*z* 46) revealed complete separation of the previously overlapping signals (Figure 2B). The analyte peak (peak 2.3) was baseline‐resolved from two coeluting components (peaks 2.2 and 2.4), confirming that the two‐dimensional setup enables resolution of compounds that are not chromatographically separated in the first dimension alone. High‐resolution Orbitrap MS analysis coupled to IRMS according to Marks et al. [21] showed the presence of three individual components and confirmed the purity of vitamin B5 prior to isotope ratio analysis (Figure S2).

These results demonstrate that heart‐cutting 2D‐LC not only mitigates matrix effects but also provides an additional level of chromatographic purification that is essential for accurate CSIA in complex supplement matrices.

### 3.3 Method Performance and Validation

Calibration experiments were performed for vitamins B5 and B9 over a concentration range of 2–100 mgC L^−1^ using external standards. The obtained performance characteristics are summarized in Table 1.

**TABLE 1:** Calibration data and isotopic performance characteristics for vitamins B5 and B9, including linearity, repeatability (SD, ‰), and mean *δ* ^13^C values of calibration standards.

Analyte	*R* ^2^	Linear range (mgC L^−1^)	MDL (mgC L^−1^)	*δ* ^13^C_mean_ (‰)	Repeatability (‰)
B5	0.9999	2–100	5	−29.25	0.11
B9	0.9999	2–100	5	−23.51	0.10

**FIGURE 3:**
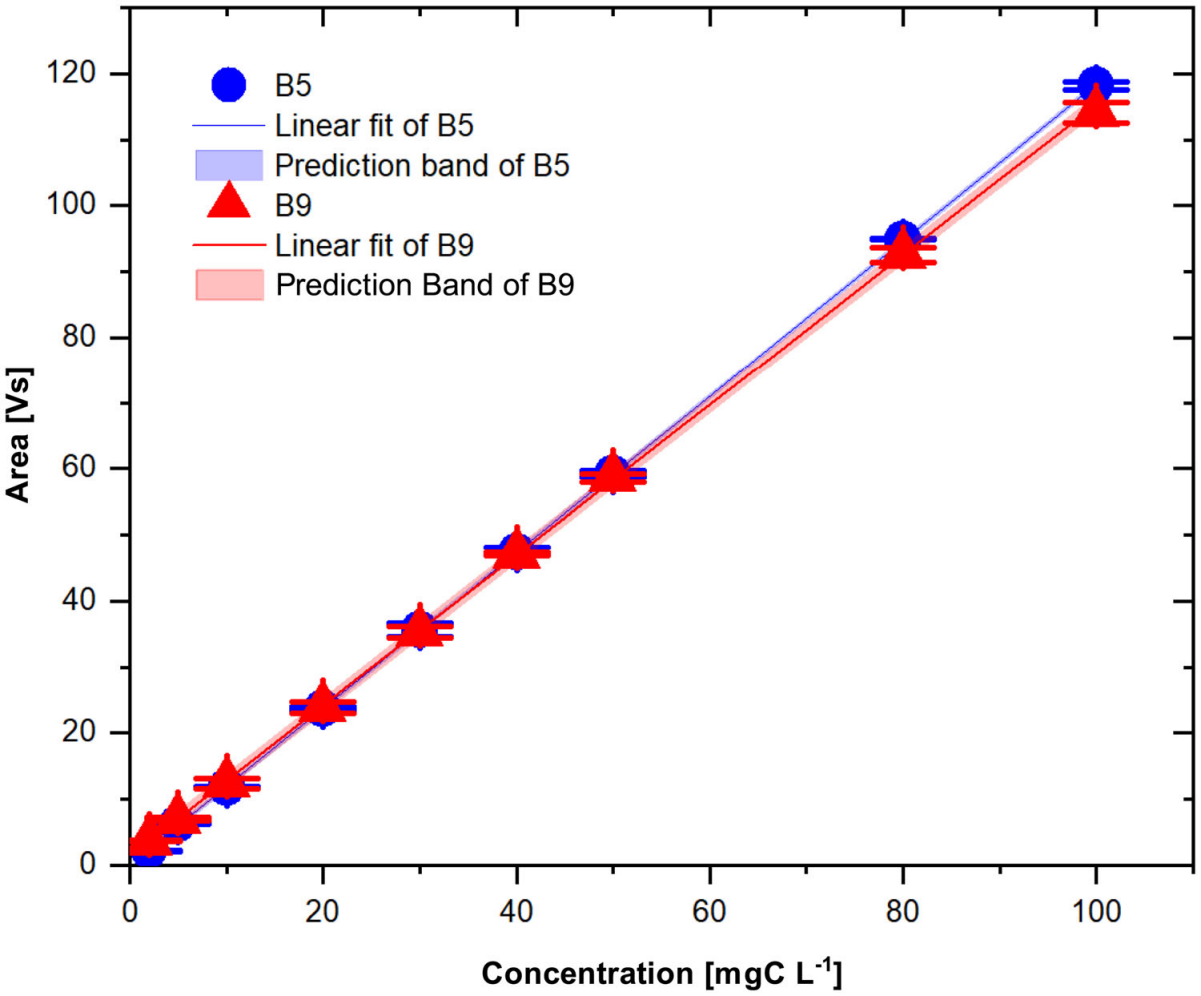
Calibration curves for vitamin B5 (blue circles) and vitamin B9 (red triangles) obtained using the optimized heart‐cutting two‐dimensional LC‐IRMS configuration. Peak area (Vs) is plotted against analyte concentration (2–100 mgC L^−1^). Each point represents the mean of *n* = 3 injections. Error bars represent ± 1 standard deviation (*n* = 3). Solid lines indicate linear regression fits.

**FIGURE 4:**
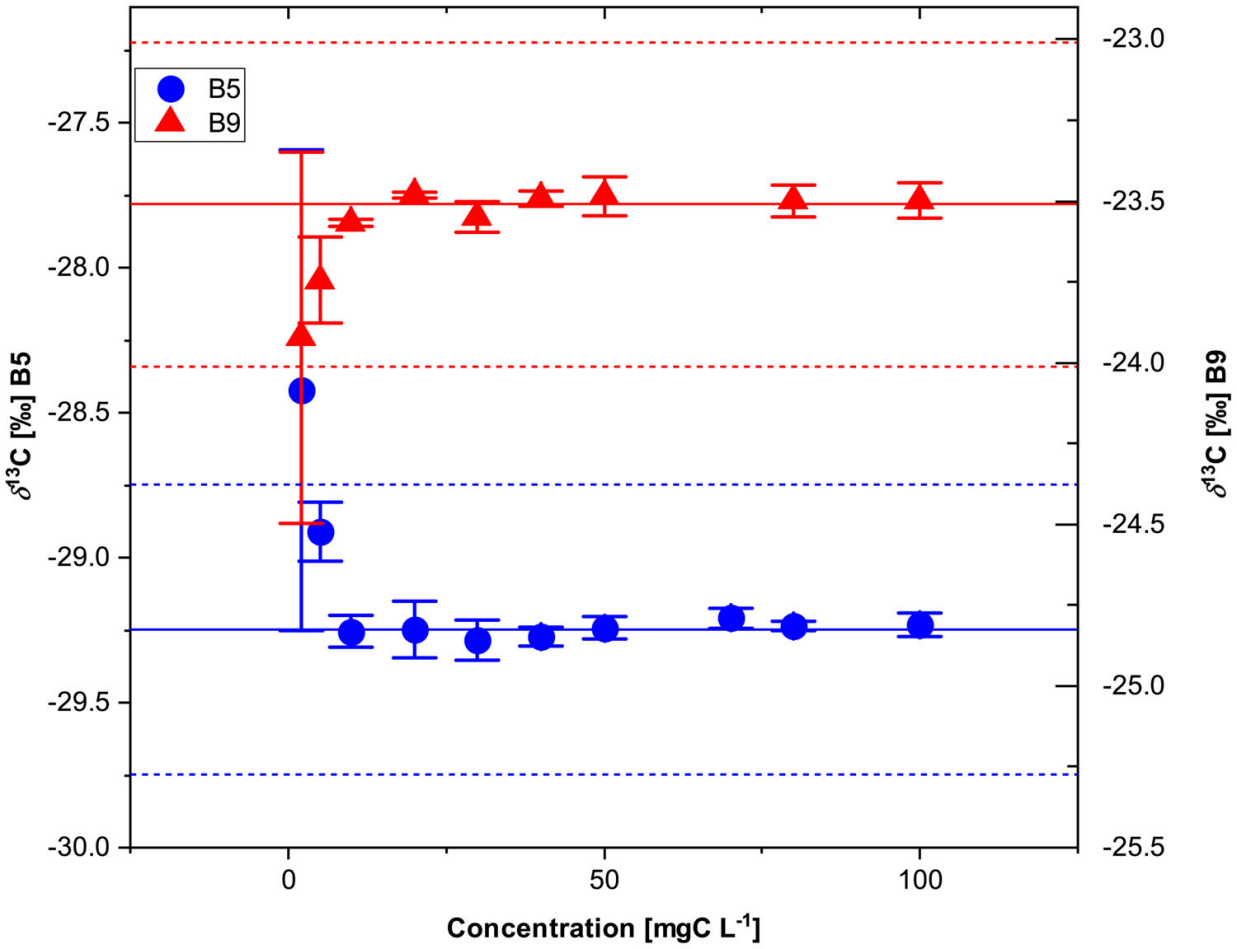
*δ* ^13^C values of vitamin B5 (blue circles) and vitamin B9 (red triangles) as a function of analyte concentration determined using the optimized two‐dimensional LC‐IRMS method. Each data point represents the mean of *n* = 3 injections. Error bars represent ± 1 standard deviation (*n* = 3). The solid line indicates the moving mean across the investigated concentration range. Dashed lines represent ± 0.5‰ deviation from the overall mean and were used as the stability criterion for method detection limit (MDL) determination.

Analyzed peak areas showed excellent linearity as a function of injected carbon amount, with correlation coefficients (*R* ^2^) of 0.9999 for both vitamins (Figure 3). No systematic trend in isotope values with concentration was observed within the investigated range, indicating absence of concentration‐dependent isotope bias (Figure 4).

Comparable calibration behavior of vitamin B5 was obtained for one‐ and two‐dimensional configurations, for both acetonitrile‐ and methanol‐based first‐dimension gradients, and for the different reversed‐phase columns tested. Statistical comparison (ANCOVA) showed no significant differences between configurations (*p* < 0.05) demonstrating that chromatographic conditions did not influence analyzed isotope ratios under the applied operating parameters.

MDLs were determined as the lowest concentration at which *δ* ^13^C values remained stable within ± 0.5‰ of the expected value. Under these criteria, MDLs of 5 mgC L^−1^ for both vitamins were obtained (Figure 4). These limits were consistent across chromatographic configurations, indicating that sensitivity was governed primarily by IRMS signal intensity rather than chromatographic mode.

Repeatability of isotope measurements was evaluated by triplicate injections of standards and selected samples. The resulting within‐sequence SDs of the triplicate measurements were ranging from 0.01‰ to 0.10‰ for vitamin B5 and 0.01‰ to 0.13‰ for vitamin B9. Instrumental stability during analytical runs was verified by repeated injections of reference gas pulses at the beginning and end of each sequence, which yielded average SDs of 0.07‰ for *δ* ^13^C values and 1.40 Vs for peak areas. All these results lie well within the typically accepted repeatability for LC‐IRMS of ± 0.5‰ and therefore confirm stable IRMS performance and demonstrate that chromatographic modulation and heart‐cutting transfer do not introduce measurable isotope variability.

Robustness of the analytical approach was assessed by comparing isotope results obtained under different chromatographic conditions, including variation of first‐dimension organic modifier (acetonitrile vs. methanol), column stationary phase in the second dimension, and dimensionality (1D vs. 2D). Across all tested configurations, analyzed *δ* ^13^C values for 100 mgC L^−1^ agreed within analytical uncertainty, with deviations below 0.05‰. This demonstrates that the isotope ratio determination is independent of chromatographic setup provided that baseline‐resolved analyte peaks are obtained prior to IRMS detection. The absence of configuration‐dependent isotope offsets confirms that the two‐dimensional approach improves chromatographic selectivity without altering isotopic integrity. Consequently, method selection can be guided by separation performance rather than isotope measurement constraints.

Overall, the validation results demonstrate that the developed 2D‐LC‐IRMS method provides precise, accurate, and configuration‐independent isotope measurements suitable for compound‐specific analysis of B vitamins in complex matrices.

### 3.4 Application to Commercial Samples

To demonstrate applicability of the developed method to real‐world matrices, a variety of commercially available vitamin‐containing products were analyzed, including tablets, effervescent formulations, powdered supplements, and beverages. These samples represent a wide range of matrix compositions and analyte concentrations, thereby providing a realistic test of method robustness.

**FIGURE 5:**
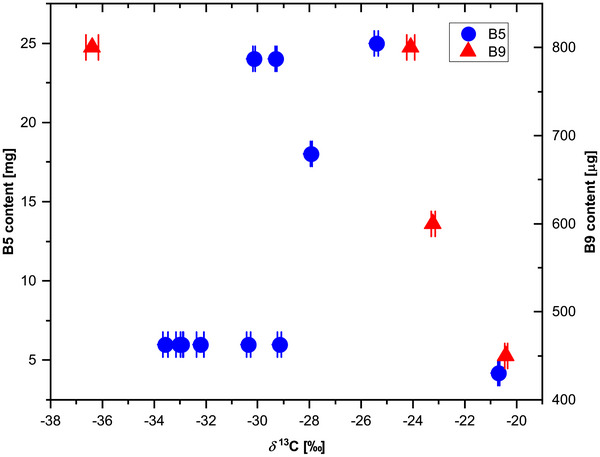
Compound‐specific *δ* ^13^C values and quantified vitamin contents of vitamins B5 (red circles) and B9 (blue triangles) in commercial dietary supplement and beverage samples (*n* = 12 products). *δ* ^13^C values were determined using the optimized heart‐cutting two‐dimensional LC‐IRMS method. Concentrations were calculated by external calibration. Error bars represent ± 1 standard deviation (*n* = 3).

Analyzed *δ* ^13^C values for vitamins B5 and B9 across all investigated samples are summarized in Figure 5 together with their contents in the samples. The analyte levels varied substantially between products, spanning 1.2–25 mg per unit for vitamin B5 and 450–800 µg per unit for vitamin B9, reflecting differences in formulation and labeling specifications. Despite this variability in concentration and matrix composition, reliable isotope ratios were obtained for all samples above the MDLs.

The *δ* ^13^C values determined for the investigated products covered a range of −20.6‰ to −32.9‰ for vitamin B5 and −20.4‰ to −36.4‰ for vitamin B9. This broad distribution indicates substantial variability in the carbon isotopic composition of the analyzed compounds across different products. Such differences are consistent with variations in synthetic pathways, precursor materials, or production processes employed by different manufacturers [22]. Notably, isotopic variability was also observed among products from the same manufacturer. For example, vitamin B5 in products from Manufacturer 1 exhibited *δ* ^13^C values ranging from −27.9‰ to −32.2‰. This intra‐brand variability may reflect differences in raw material sourcing, supplier changes, batch‐to‐batch production variations, or a combination of these factors, all of which can influence the carbon isotopic signature of the final compound [22, 23].

The combined determination of concentration and isotope composition within a single analytical workflow demonstrates the versatility of the method and highlights its potential for future applications in authenticity assessment, quality control, and investigation of synthetic production routes. These results illustrate that the developed approach is broadly applicable to complex commercial products, requiring only minor adjustments such as extraction volume and target concentration rather than fundamental changes to the chromatographic method.

## 4 Conclusions

The results demonstrate that reliable isotope ratios can be obtained for B vitamins in diverse commercial products, despite substantial differences in formulation composition and analyte concentration. The two‐dimensional setup provided effective removal of matrix or coeluting components, improved chromatographic peak quality, and stable isotope measurements without introducing isotope fractionation or configuration‐dependent bias. Method performance was consistent across chromatographic conditions, confirming that isotope determination is governed primarily by instrumental detection rather than separation mode when sufficient chromatographic resolution is achieved.

The presented strategy extends the analytical scope of LC‐IRMS to a compound class that has not previously been accessible to this technique and establishes a general workflow for isotope analysis of polar, nonvolatile analytes in complex matrices. Beyond vitamin analysis, the approach is applicable to a wide range of compounds where matrix interference and mobile‐phase restrictions currently limit compound‐specific isotope measurements. Future work may focus on expanding the method to additional vitamins and micronutrients, integration with multidimensional chromatographic selectivity concepts, and application to authenticity, traceability, and production pathway studies. The results therefore provide both a practical analytical solution and a methodological framework for advancing LC‐IRMS applications in food, pharmaceutical, and environmental analysis.

## Author Contributions

**Sarah P. Rockel**: conceptualization, investigation, visualization, methodology, data curation, formal analysis, writing – original draft, writing – original draft preparation. **Jaqueline Martiny**: investigation, writing – review and editing. **Maik Jochmann**: supervision, writing – review and editing. **Torsten C. Schmidt**: supervision, writing – review and editing.

## Conflicts of Interest

The authors declare no conflicts of interest.

## Supporting information




**Supporting File**: jssc70419‐sup‐0004‐SuppMat.docx.

## Data Availability

The data that support the findings of this study are available from the corresponding author upon reasonable request.
