# Supplementary material for: Heart‐Cutting Two‐Dimensional Liquid Chromatography‐Isotope Ratio Mass Spectrometry for Compound‐Specific δ 13C Analysis of Water‐Soluble B Vitamins in Complex Supplement Matrices
Source: J Sep Sci. 2026 Apr 21;49:e70419. doi: 10.1002/jssc.70419 (PMC13099010; doi:10.1002/jssc.70419)
Supplement: Supplementary file 1 — Supporting File: jssc70419‐sup‐0004‐SuppMat.docx. [file JSSC-49-e70419-s001.docx]

**Supplementary Material**

**Heart-Cut Two-Dimensional Liquid Chromatography-Isotope Ratio Mass Spectrometry for Compound-Specific *δ*^13^C Analysis of Water-Soluble B Vitamins in Complex Supplement Matrices**

Sarah P. Rockel^a,b^, Jacqueline Martiny^a,c^, Maik A. Jochmann^a,*^, Torsten C. Schmidt^a,b^

^a^Instrumental Analytical Chemistry, University of Duisburg-Essen, Universitätsstraße 5, 45141 Essen, Germany

^b^Centre for Water and Environmental Research (ZWU), University of Duisburg-Essen, Universitätsstraße 2, 45141 Essen, Germany

^c^ ^c^MDS Holding GmbH & Co. KG, Kirchhörder Straße 29, 44229 Dortmund; Germany

Corresponding author: [sarah.rockel@uni-due.de](mailto:sarah.rockel@uni-due.de)

**Table S1:** Overview of commercial vitamin samples analysed in this study

| **Sample** | **Product name** | **Manufacturer** | **Product type** |
| --- | --- | --- | --- |
| 1 | A-Z complex | M1 | Film-coated tablet |
| 2 | Vitamin B plus | M1 | Film-coated tablet |
| 3 | B complex forte mini | M1 | Film-coated tablet |
| 4 | Multi complex A-Zink | M1 | Film-coated tablet |
| 5 | Hydration sticks | M2 | Powder |
| 6 | Multivitamin effervescent tablet | M3 | Effervescent tablet |
| 7 | Magnesium effervescent tablet | M3 | Effervescent tablet |
| 8 | Vitamin B complex | M4 | Capsule |
| 9 | Rehab | M5 | Energy Drink |
| 10 | Folic acid 800 + B vitamins | M6 | Film-coated tablet |
| 11 | A-Z complete depot | M6 | Film-coated tablet |
| 12 | Multivitamin effervescent tablet | M7 | Effervescent tablet |

**S1: Comparison of impact of organic modifiers in the first chromatographic dimension on second dimension separation**

**Figure S1:** Comparison of impact of organic modifiers in the first chromatographic dimension on second dimension separation. (A) Methanol (MeOH) and (B) acetonitrile (ACN) as organic modifiers. Second dimension chromatograms (mass traces 44, 45, and 46) are shown for the transfer of vitamin B5.

To evaluate the influence of the organic modifier in the first chromatographic dimension, separations were performed using both acetonitrile (ACN) and methanol (MeOH). As shown in Figure S1, the use of MeOH (Panel A) resulted in a substantially higher organic background signal after heart-cut transfer to the second dimension. The elevated background partially saturated the IRMS detector and required an extended stabilization period before baseline conditions were re-established. In addition, peak broadening was observed under MeOH conditions, resulting in reduced peak sharpness and less defined peak boundaries. In contrast, the use of ACN (Panel B) led to significantly lower organic background transfer to the second dimension. Baseline stabilization occurred more rapidly, and chromatographic peaks exhibited improved symmetry and narrower peak widths. The resulting sharper peak profiles facilitated more reliable peak integration and enabled shorter overall method runtimes.

Based on these observations, ACN was selected as the preferred organic modifier for analysis of commercial samples. However, isotope ratio measurements obtained using MeOH did not show systematic deviations, indicating that both modifiers are in principle compatible with the 2D-LC-IRMS configuration.

**S2: High-resolution mass spectrometric characterization of coeluting compounds**

To further investigate the coeluting signals observed in the one-dimensional chromatographic separation, selected fractions were analysed using high-resolution mass spectrometry (Orbitrap HRMS) in parallel with isotope ratio mass spectrometry (IRMS) according to Marks et al. (2022)

Figure S2 shows the total ion chromatogram (TIC) obtained for the investigated sample, together with extracted ion chromatograms (EICs) at m/z 220.1174, 83.0856, and 181.9907. The signal at m/z 220.11 was assigned to protonated pantothenic acid (vitamin B5) and co-eluted with additional mass traces under one-dimensional conditions. The extracted ion chromatograms demonstrate that, following heart-cut transfer into the second dimension, chromatographic separation allowed selective isolation of pantothenic acid prior to IRMS detection. The HRMS data therefore confirm that the apparent single peak in the first dimension represents a coelution of at least three components.

**Figure S2:** Total ion chromatogram (TIC) and extracted ion chromatograms (EICs) at m/z 220.11 (pantothenic acid), 83.085, and 184.01 for the investigated multivitamin effervescent tablet sample after second dimension separation.
